# Health literacy in a community with low levels of education: findings from Chakaria, a rural area of Bangladesh

**DOI:** 10.1186/s12889-017-4097-y

**Published:** 2017-02-16

**Authors:** Susmita Das, Mohammad Nahid Mia, Syed Manzoor Ahmed Hanifi, Shahidul Hoque, Abbas Bhuiya

**Affiliations:** 1 Health Systems and Population Studies Division, International Centre for Diarrhoeal Disease Research, Bangladesh (icddr,b), Dhaka, Bangladesh; 2 Partners in Population and Development, Dhaka, Bangladesh

**Keywords:** Health literacy, Hypertension, Diabetes, Village doctor

## Abstract

**Background:**

Health literacy (HL) helps individuals to make effective use of available health services. In low-income countries such as Bangladesh, the less than optimum use of services could be due to low levels of HL. Bangladesh’s health service delivery is pluralistic with a mix of public, private and informally trained healthcare providers. Emphasis on HL has been inadequate. Thus, it is important to assess the levels of HL and service utilization patterns. The findings from this study aim to bridge the knowledge gap.

**Materials and Methods:**

The data for this study came from a cross-sectional survey carried out in September 2014, in Chakaria, a rural area in Bangladesh. A total of 1500 respondents were randomly selected from the population of 80,000 living in the Chakaria study area of icddr, b (International Centre for Diarrhoeal Disease Research, Bangladesh). HL was assessed in terms of knowledge of existing health facilities and sources of information on health care, immunization, diabetes and hypertension. Descriptive and cross-tabular analyses were carried out.

**Results:**

Chambers of the rural practitioners of allopathic medicine, commonly known as ‘village doctors’, were mentioned by 86% of the respondents as a known health service facility in their area, followed by two public sector community clinics (54.6%) and Union Health and Family Welfare Centres (28.6%). Major sources of information on childhood immunization were government health workers. Almost all of the respondents had heard about diabetes and hypertension (97.4% and 95.4%, respectively). The top three sources of information for diabetes were neighbours (85.7%), followed by relatives (27.9%) and MBBS (Bachelor of Medicine and Bachelor of Surgery) doctors (20.4%). For hypertension, the sources were neighbours (78.0%), followed by village doctors (38.2%), MBBS doctors (23.2%) and relatives (15%). The proportions of respondents who knew diabetes and hypertension control measures were 40.9% and 28.0%, respectively. More females knew about the control of diabetes (44.4% to 36.6%) and hypertension (31.1% to 24.2%) than males.

**Conclusions:**

A low level of HL in terms of modern health service facilities, diabetes and hypertension clearly indicated the need for a systematic HL programme. The relatively high levels of literacy concerning immunization show that it is possible to enhance HL in areas with low levels of education through systematic awareness-raising programmes, which could result in higher service coverage.

## Background

Very simply put, Health Literacy (HL) is defined as the “ability to engage with health information and services” [1]. The phrase “Health Literacy” has evolved as a dynamic multi-layered concept since its appearance in the literature in 1974 [2]. HL refers to multiple factors that individuals or communities need to access, understand, appraise and use health-related information and services for making the best health-related decisions [1, 3]. These factors include individual characteristics, general literacy, experience with diseases and health systems, and social, cultural, and psychological factors [1, 3]. In today’s societies, HL is gaining more attention than before for many reasons. Modern health systems are complex and can be difficult to navigate and understand. The education systems might not equip people with necessary skills that will help them effectively interact with modern health systems and information for improving their health [4, 5]. Differences in HL contribute to health inequities and health outcomes [1]. There are mainly three types of HL: basic/functional, interactive/communicative and critical HL [6]. Basic HL refers to the minimum amount of reading and writing skills necessary for everyday functioning, whereas interactive HL includes higher-level cognitive, literacy and social skills that enable people to extract, interpret and use information accordingly [6]. Critical literacy is associated with higher autonomy and empowerment as it allows for the critical analysis of information and greater control over situations [6]. Low HL leads to a detrimental lifestyle and behaviour; prevents the uptake of disease prevention and detection services; hampers self-management of chronic disease, compliance with medications and understanding of provider communication; raises health care costs; and worsens existing inequities [4]. Non-communicable diseases (NCDs) are one of the leading causes of deaths globally and are associated with multiple modifiable behavioural risk factors [4, 7]. Low HL adversely affects the behavioural risk factors of NCDs and is often worse for vulnerable groups such as the elderly, individuals with low levels of education, minorities and immigrants, who have inadequate HL as well as a high risk for developing NCDs [4]. NCDs are responsible for 59% of the proportional mortality in Bangladesh (% of the total deaths in both sexes and all age groups) [8]. In 2011, the BDHS (Bangladesh Demographic and Health Survey) report stated that 32% of women and 19% of men had elevated blood pressure (BP) and an additional 28% of women and men were pre-hypertensive; 11% of men and women were diabetic and 25% of women and men were pre-diabetic [9]. The struggling health system is inadequately prepared to face this challenge [10]. Bangladesh already has a critical shortage of health workers with urban clustering [11]. Despite having a public health delivery system with a vast network of public health facilities and providers arranged according to administrative tiers, informal providers are trusted health care providers for the many rural people of Bangladesh for health services and information [11].

There have not been adequate systematic explorations of HL in Bangladesh. In the context of rural Bangladesh, this study attempts to fill this knowledge gap. In our study, we considered HL in terms of knowledge of existing health facilities, care-seeking patterns, immunization, and knowledge of diabetes and hypertension.

## Materials and Methods

### Study Area

The study was conducted in Chakaria Upazila (a sub district) under the Cox’s Bazar District situated in the southeast coastal area of Bangladesh [12, 13]. Like other parts of Bangladesh, agricultural farming is one of the major economic activities of the villagers, along with woodcutting and fishing, due to the proximity of the villages to the sea and forest. Thirty percent of the households were landless and about half of the households were dependent on income from menial labour [14]. The study site has been one of the most conservative areas in terms of religion and openness to modern ideas, with low levels of secular education. The literacy rate among the population aged 7 years or older was 47.0% and was similar among males and females [15]. More than 90% percent of the population were Muslim, while the remainder were either Hindu or Buddhist [14, 16]. At the time of this survey, there was one Upazila Health Complex at the sub-district level, 10 Union Health and Family Welfare Centres (UHFWCs) at the union level, 254 satellite clinics for immunization run by government health workers, 23 Community Clinics at the lowest level and one rural dispensary for first-level services run by the government. In addition, there were 7 community-established and icddr,b facilitated Village Health Posts for primary care consultation services, and 3 private diagnostic centres [13]. Apart from these, 342 village doctors (informal) provided primary care and dispensed both allopathic and homeopathic drugs [13]. In 2012, the life expectancy of the population at birth in the study area was 68 years for males and 70 years for females [13]. In Chakaria, pulmonary tuberculosis, acute respiratory infection, stroke, diabetes and chronic obstructive pulmonary diseases were the leading causes of death in all age groups [14].

### Study design and data collection

This study is a cross-sectional survey carried out in some villages of Chakaria, where icddr,b has been running a Health and Demographic Surveillance System (HDSS) comprising a population of 80,000. In September 2014, a total of 1500 respondents, with equal proportions of males and females, aged more than 18 years were randomly selected from the Chakaria HDSS database. Of the 1500 respondents, 283 could not be interviewed due to their non-availability; 23 migrated out of the area, 22 died, 226 were absent at the time of the visit, 5 were deaf, 3 were sick, and 4 respondents refused to be interviewed. Thirteen local female interviewers with at least 10 years of schooling were recruited for data collection. The interviewers received a two-day training on interviewing before data collection. The questionnaire was drafted in *Bangla* and developed on the basis of previous literature and available information in the context of the health system, e.g., the existing health facilities and the existing health care system of Bangladesh. The questionnaire had some listed answers with provisions for recording other answers. For some questions, provisions for recording multiple answers were made. Two experienced supervisors supervised the data collection operation. The supervisors revisited 5% of the randomly chosen respondents within 2 days of data collection by the field workers to ensure the data quality. Later, the supervisors and the field workers sorted out any inconsistencies in the data collected during the re-interviews. All completed questionnaires were manually checked for completeness and for any inconsistencies, which were identified by computer-based ranges and consistency checks.

### Definition of the study variables

HL was assessed by the knowledge of existing health facilities, immunization, diabetes and hypertension detection and control measures and the common consultation of health care providers. The respondents were asked the following questions: “Please name the health facilities that exist in your union?”, “Whom do you usually consult when you need any information about health-related matters? Please mention the first three people in order of priority.”; “Has s/he (child less than five years of age) been vaccinated?”; “How did you know for the first time whether you are diabetic or not?” “How can diabetes be controlled? (Multiple answers possible)”; “How did you know for the first time whether you are hypertensive or not?”; “How can hypertension be controlled? (Multiple answers possible)”. The mode of identifying diabetes was categorized as urine and/or blood test and others, and mode of detecting hypertension were categorized as a blood-pressure measurement by a health care provider or by other means. Where appropriate, multiple responses were recorded. The respondents’ background characteristics, including age, number of completed years of schooling, and a list of household items owned by any member of the household were recorded. Items owned by the household were used to calculate a household asset score using Principal Component Analysis (PCA) [12, 17].

### Data analysis

Descriptive and cross-tabular analyses were carried out using STATA software (STATA/SE version14; StataCorp, 4905 Lakeway Drive, College Station, Texas 77845 USA). Chi–square and Fisher’s exact tests were performed to assess the statistical significance of associations between the outcome variables and the background characteristics of the respondents.

## Results

The survey team interviewed 1217 respondents out of 1500 sampled respondents through repeated visits. Three visits were made to minimize the rate of non-response. Men were less frequently available for the interview during daytime due to their external engagements. In total, 72.1% (541) males and 90.1% (676) females were interviewed.

### Background characteristics of the respondents

Forty-four percent of the 1217 people interviewed were males. Approximately 8% of the respondents were between 20 and 29 years of age, and 24% were 60 years old or older. One-fourth of the respondents were between 30 and 39 years of age, and 22% and 20% were between 40 and 49 years and 50 and 59 years of age, respectively. Fifty-two percent of the respondents had no formal schooling, while 29% had 1–5 years of schooling; only 19% had 6 or more years of schooling.

### Knowledge of existing health service facilities

Table 1 presents the respondents’ level of knowledge of existing health facilities in the study area. The village doctor’s chamber was the most widely known service facility (86.0% of respondents and 46.0% of responses), followed by the community clinic (54.6% of respondents and 29.2% of responses), the Union Health and Family Welfare Centre (28.6% of respondents and 15.3% of responses), and icddr,b-run village health posts (12.3% of respondents and 6.6% of responses).

**Table 1:** Knowledge of existing health facilities among the respondents^a^

Facilities	% of the responses	% of respondents
Village doctor's chamber	45.9	86.0
Community clinic	29.2	54.6
Union Health and Family Welfare Centre (UHFWC)	15.3	28.6
icddr,b health centre	6.6	12.3
SACMO’s (Sub assistant community medical officer) chamber	0.9	1.7
Others	2.1	3.9
N	2279	1217

^a^Multiple responses were reported by a given respondent, therefore the number of responses are higher than number of respondents

### Preferred sources of consultation for health matters and use

Graduate physicians or formally trained registered medical practitioners with MBBS degrees were the most preferred source for consultations on health matters (41.6%), followed by village doctors (37.5%) and family members (15.1%). More females than males preferred to consult family members for health-related matters. Males were more likely to prefer consulting sources outside of the family, e.g., MBBS doctors, SACMO and village doctors, for health-related matters. With the improvement of socioeconomic status and education levels, more respondents preferred to consult MBBS doctors for health matters and the preference for village doctors declined (Table 2).

**Table 2:** First preference health care information providers by socio-demographic characteristic

Socio-demographic Characteristics	No. of respondents	Family	Neighbour	Drug seller/Village doctor	MBBS doctor	SACMO	Others	*P*-value
All	1217	15.1	0.8	37.5	41.6	3.6	1.4	
Sex								0.013
Male	541	11.3	0.9	38.5	44.6	4.3	0.9	
Female	676	18.2	0.7	36.7	39.2	3.1	1.8	
Age								0.436
20–29	102	21.6	0.0	40.2	32.4	2.9	2.9	
30–39	306	12.8	0.7	41.2	38.2	5.6	1.3	
40–49	273	14.3	1.1	37.4	42.1	3.7	1.8	
50–59	245	17.1	0.8	31.8	46.9	2.5	0.4	
60+	291	14.4	1.0	37.5	43.3	2.8	1.4	
Education								0.002
None	635	14.7	1.1	42.5	36.4	4.1	0.9	
1–5 years	355	13.2	0.6	34.6	46.5	3.1	2.3	
6+ years	227	19.4	0.4	27.8	48.4	3.1	1.3	
Asset quintile								<0.001
Lowest	246	15.0	0.4	48.4	31.3	2.9	2.0	
2nd	242	10.3	0.0	43.0	37.2	8.3	1.2	
Middle	244	11.1	1.6	43.0	41.4	2.5	0.8	
4th	242	16.5	1.7	30.6	46.7	2.9	0.8	
Highest	243	22.6	0.4	22.2	51.4	0.8	2.1	

Table 3 presents the source of consultation in the case of sickness during the last twelve months. In as many as 51.6% of the cases, the respondents contacted a village doctor followed by MBBS doctors (39.4%) and SACMO (4.9%). Males and females consulted MBBS doctors equally, while more males than females sought consultation from village doctors and SACMO. Respondents with higher education levels and socioeconomic status were more likely to seek consultations from MBBS doctors, and this trend was statistically significant. The opposite was observed for village doctors.

**Table 3:** Health care provider consultation in the last year by socio- demographic characteristics

Socio-demographic Characteristics	No. of respondents	Consulted with drug seller/Village doctor	MBBS doctor	SACMO	Health and family planning worker	Others	*P*-value
All	987	51.6	39.4	4.9	1.9	2.2	
Sex							0.027
Male	404	53.5	39.6	5.2	0.5	1.2	
Female	583	50.3	39.3	4.6	2.9	2.9	
Age							0.003
20–29	77	51.9	32.5	9.1	5.2	1.3	
30–39	237	59.9	29.5	7.2	2.1	1.3	
40–49	221	50.7	43.4	3.6	1.4	0.9	
50–59	203	47.3	42.4	4.4	1.5	4.4	
60+	249	47.8	45.0	2.8	1.6	2.8	
Education							<0.001
None	525	56.6	33.9	4.6	1.7	3.2	
1–5 years	300	49.7	40.7	6.0	2.0	1.6	
6+ years	162	38.9	54.9	3.7	2.5	0.0	
Wealth index							<0.001
Lowest	197	64.0	24.9	5.6	1.5	4.0	
Second	202	59.9	29.2	6.4	4.0	0.5	
Middle	197	53.3	38.1	5.1	2.5	1.0	
Fourth	196	52.0	38.8	5.1	0.5	3.6	
Highest	195	28.2	66.6	2.1	1.0	2.1	

### Immunization

Out of the total of 550 under-five children residing in the respondents’ households, 97.8% were reported to be vaccinated. Out of the total respondents, 45.4% learned about vaccination from EPI (Expanded Programme on Immunization) workers, 40.7% from non-EPI health workers, 19.5% from neighbours and 13.0% from loudspeaker announcements by EPI workers (Table 4).

**Table 4:** Distribution of sources of information regarding childhood immunization^a^

Sources of information	% of responses	% of respondents
EPI worker	35.0	45.4
Health worker	31.4	40.7
Neighbours/villagers	15.0	19.5
Miking (loud speaker)	10.0	13.0
Family member	2.6	3.4
Book	1.4	1.9
Others	4.6	5.9
N	698	538

^a^Multiple responses were reported by a given respondent, therefore the number of responses are higher than number of respondents

### Diabetes and hypertension

The majority of the respondents had heard about diabetes (97.4%) and hypertension (95.4%). Slightly higher proportions of males compared to females knew about diabetes (99.1% versus 96.0%) and hypertension (97.2% versus 93.9%). A total of 1989 responses were recorded for diabetes and 1976 for hypertension.

The most frequently mentioned source of information for diabetes was neighbours (85.7%), followed by relatives (27.9%), MBBS doctors (20.4%) and village doctors (18.5%) (Table 5). Fewer respondents in the low socioeconomic status group (12.5%), the illiterate group (14.7%), and females (20.0%) reported that formal healthcare providers such as MBBS doctors were their source of information compared to respondents from the high socioeconomic status group (34.3%), those with 6 or more years of education (30.4%), and males (20.9%). The proportion of respondents reporting family members as their source of information about diabetes increased from 8.6% among illiterates to 13.2% among those who had more than 6 years of education.

**Table 5:** Sources of information on diabetes by socio- demographic characteristics^a^

Socio-demographic characteristics	No. of respondents	Family	Relatives	Neighbour	Drug seller/Village Doctor	MBBS doctor	Others	*P*-value
All	1185	9.8	27.9	85.7	18.5	20.4	5.6	
Sex								<0.001
Male	536	7.5	21.8	86.4	23.0	20.9	8.4	
Female	649	11.7	32.8	85.2	14.8	20.0	3.2	
Age (years)								0.143
20–29	102	10.8	34.3	85.3	19.6	15.7	3.9	
30–39	301	7.0	29.2	87.7	19.9	17.3	7.3	
40–49	268	8.2	28.0	86.6	18.7	18.3	3.4	
50–59	242	7.4	26.3	87.2	21.5	26.4	6.6	
60+	272	16.2	25.4	81.6	13.6	22.4	5.5	
Education								<0.001
None	605	8.6	24.6	87.9	17.8	14.7	4.0	
1–5 years	353	9.6	27.8	87.0	17.3	23.8	3.7	
6+ years	227	13.2	36.6	78.0	22.0	30.4	12.9	
Wealth index								<0.001
Lowest	232	3.5	22.4	91.4	23.3	12.5	4.3	
Second	233	4.7	22.7	94.0	10.2	11.6	5.6	
Middle	239	6.3	27.2	85.4	15.5	20.1	5.4	
Fourth	239	13.0	31.8	81.6	20.1	23.0	5.4	
Highest	242	21.1	34.7	76.9	13.6	34.3	7.0	

^a^Multiple responses were recorded

Table 6 shows the likely sources of information about hypertension. Neighbours were the most important source of information about hypertension irrespective of age, sex, education and household socioeconomic status. The proportion of respondents seeking information from MBBS doctors was higher among respondents with 6 or more years of education and those with high socioeconomic status compared to those with no education (16.1% versus 36.3%) and low socioeconomic status (10.0% versus 39.7%). The prevalence of family members and relatives as a source of information increased with the respondents’ socioeconomic status (9.1% versus 19.7%; 13.9% versus 16.7%) and education (11.2% versus 13.9%; 14.2% versus 19.3%).

**Table 6:** Sources of information on hypertension by socio demographic characteristics^a^

Socio-demographic characteristics	No. of respondents	Family	Relatives	Neighbour	Drug seller/Village Doctor	MBBS doctor	Others	*P*-value
All	1161	12.2	15.0	78.0	38.2	23.2	3.6	
Sex								0.002
Male	526	12.2	11.4	77.9	44.3	23.0	4.4	
Female	635	12.3	18.0	78.1	33.1	23.3	2.7	
Age (years)								0.249
20–29	100	13.0	19.0	79.0	34.0	20.0	3.0	
30–39	292	9.3	16.8	82.9	40.1	17.5	1.7	
40–49	262	11.5	14.5	76.3	40.5	25.2	3.1	
50–59	239	13.0	15.1	73.2	42.3	27.2	6.7	
60+	268	15.3	11.9	78.4	31.7	25.0	3.7	
Education								<0.001
None	590	11.2	14.2	78.8	39.2	16.1	2.9	
1–5 years	348	12.9	13.5	79.3	37.4	26.7	2.9	
6+ years	223	13.9	19.3	74.0	36.8	36.3	6.7	
Wealth index								<0.001
Lowest	230	9.1	13.9	80.9	47.4	10.0	0.9	
Second	229	10.0	12.7	83.4	39.7	16.2	3.9	
Middle	235	10.6	11.9	75.7	38.3	23.4	3.8	
Fourth	233	11.6	19.7	77.3	36.1	26.2	3.0	
Highest	234	19.7	16.7	73.1	29.5	39.7	6.4	

^a^Multiple responses were recorded

Interestingly, for both diabetes (85.7%) and hypertension (78.0%), neighbours were the most frequently reported source of information followed by drug sellers/village doctors (18.5% and 38.2%), relatives (27.9% and 15.0%), MBBS doctors (20.4% and 23.2%), and family members (9.8% and 12.2%) (Tables 5 and 6).

### Diabetes and hypertension among respondents

A total of 1185 respondents had heard about diabetes. Among them, only 5.7% reported that they were diabetic, while 79.8% claimed to be non-diabetic and 14.5% did not know whether they were diabetic or not. Sixty-nine percent of the identified diabetics learned of their status through blood and/or urine tests. Nearly one-third of the diabetics were informed about their disease by another source (Table 7).

**Table 7:** Distribution of modes of detecting diabetes among respondents

Mode of detection	Male	Female	Total	*P*-value
Blood test and/or Urine test	68.4	69.6	69.3	0.973
Symptoms, told by doctor	31.6	30.4	30.7	
No. of respondents	17	51	68	

Out of 1161 respondents who had heard about hypertension, the majority (71.1%) reported not being hypertensive and only 11.2% reported being hypertensive, while the rest (17.7%) did not know whether they were hypertensive or not. Ninety-seven percent of the hypertensive respondents learned of their diagnosis through a blood pressure (BP) measurement by a health care provider and the rest learned of their diagnosis from suspected symptoms. The differences observed in the mode of detecting hypertension between males and females were not statistically significant (*p* = 1.00) (Table 8).

**Table 8:** Distribution of modes of detecting hypertension among respondents

Mode of detection	Male	Female	Total	*P*-value
Measurement of BP by any provider	96.4	97.1	96.9	1.00
Suspected because of symptom	3.6	2.9	3.1	
No. of respondents	28	102	130	

### Control of diabetes and hypertension

In total, 44.4% (300 out of 676) of females and 36.6% (198 out of 541) of males knew about control measures for diabetes (*p* < 0.001). Among them, 73.1% of respondents mentioned a low-carbohydrate diet, physical activity or exercise, drugs, lowering anxiety, low-fat food and the avoidance of sweetened foods as measures to control diabetes, with similar levels of knowledge among males and females (Table 9).

**Table 9:** Diabetes control measures by sex of the respondents

Mentioned control measure	Male	Female	Total	*P*-value
Low carbohydrate diet, Physical activity or exercise, Drugs, Reducing anxiety, Eating low fat food, Avoiding sweetened food	72.7	73.4	73.1	0.781
Eating more vegetables, stop eating when stomach is partially full, quitting smoking	27.3	26.6	26.9	
Number of respondents	198	300	498	

In total, 24.2% (131 out of 541) of males and 31.1% (210 out of 676) of females knew about measures to control hypertension (*p* < 0.001). Among them, 79.4% of respondents mentioned drugs, physical exercise, anxiety reduction, reduced salt consumption, and nutritious food as methods for controlling hypertension. A slightly higher percentage of males (82.1%) compared to females (77.8%) mentioned these measures for controlling hypertension (Table 10).

**Table 10:** Hypertension control measures by sex of the respondents

Mentioned control measure	Male	Female	Total	*P*-value
Low fat diet, Physical exercise, Decrease food intake, Lower anxiety, Reduce salt consumption, Eating nutritious food	82.1	77.8	79.4	0.249
Resting and seeking advice from doctor	17.9	22.2	20.6	
Number of respondents	131	210	341	

## Discussion

Health decision making is a dynamic interaction influenced by an individual’s ability to access, appraise, understand and decide while interacting with information, systems, support, resources and the environment. This interaction is driven by the “contextual demand” of the specific disease, communication characteristics of the practised medical culture, the health care system and society’s value of its members. Thus, HL is context- and content-specific [1].

### Health facility and care seeking

In Bangladesh, the archetypal public sector health service in rural areas is distributed through a vast network of a multi-tier public health facilities and providers arranged according to administrative level (national, divisional, district, upazila/sub district, union and ward). Primary health care is delivered through upazila and lower down public health facilities, while district or upper-level facilities provide secondary and tertiary care [18]. Despite the presence of formal providers from public and private sectors, “informal providers” provide care to the majority of the Bangladeshi rural population due to the poor quality of the public health care and scarce resources and facilities [11]. “Informal providers” are not registered with any government regulatory body and can be found in both sectors [19]. They include community health workers (CHWs), village doctors, drug vendors, traditional healers, faith healers, traditional birth attendants, and homeopaths, among others [19]. They practise allopathic, homeopathic, and traditional medicine with little or no formal training in medicine. The informal providers are close to the community’s homes and are rooted in their belief system, despite the presence of formal providers [19]. Our results also support this fact (Table 1). Despite the presence of both state and privately run facilities, the chamber of the local village doctor, who is an informal provider, was the most widely known health care facility among the respondents. Earlier studies from Chakaria reported that as much as 96% of the all health care providers were informal [20]. In terms of health care seeking, in actual practice, nearly half of the respondents consulted village doctors despite stating a preference for MBBS doctors (Table 3). A study performed in Chakaria in 2007 reported that consultations with village doctors (65%), irrespective of disease types, were much more frequent compared to consultations with MBBS doctors and other types of health care providers (14%) [21]. In our study, we also found that a higher proportion of respondents received consultations from informal healthcare providers, i.e., village doctors, but consultations from MBBS doctors had risen to 39%. With the rise in education level and socio economic status, more people went to MBBS doctors and fewer went to village doctors. Interestingly, males and females consulted MBBS doctors with the same frequency in this study.

Informal providers comprise 51%-96% of providers in developing countries such as Bangladesh, India, and Uganda due to their convenience, affordability and cultural acceptability [22]. Although they may provide questionable and even harmful care, village doctors are a trusted source of care and are intricately embedded in the social fabric in Bangladesh [23]. With the critical shortage in the health workforce and the epidemiological transition at hand, non-physician health care provider empowerment and task shifting are favoured for improving access and coverage [24]. Bangladesh itself has successfully used village doctors in its tuberculosis control programme to refer suspected cases and to distribute DOTS therapy [25]. China’s “bare foot doctors” were systematically trained for a short duration to provide basic curative services and health education to rural populations [26].

### Immunization

Bangladesh has admirably reduced child mortality. Childhood immunization through EPI is a major catalyst in this success. According to recent national data, 78% of 12- to 23-month-old children were vaccinated with all recommended vaccines before their first birthday [27]. In our study, we found that knowledge about childhood vaccination was universal among the respondents. Their source of knowledge is mostly public sector grass root health workers, with much smaller contributions from family and community members. Another study conducted in a rural area of Bangladesh also reported that the public sector health workers are the major source of immunization information for the rural masses [28]. Undoubtedly, these grass roots-level health workers, such as health assistants, are the driving force of the successful EPI campaign and the source of immunization information. This issue is an important point to consider because a shortage of this crucial health force can impede the progress of the programme. In 2013, 16.7% of the sanctioned posts for domiciliary staff (health inspectors, assistant health inspectors, and health assistants) were vacant [18]. Understanding only the benefit of immunization is not going to be useful for ensuring full immunization coverage [29]. Immunization can benefit children when the full schedule is completed with the right doses administered at the right times. Health workers play a very important role in driving this message home. Communities’ reliance on community health workers for vaccinating their children is thought to be more important than the physical existence of health care facilities [30].

### Diabetes and hypertension

Despite making progress in maternal and child health and in a few communicable diseases, Bangladesh is facing a new threat from NCDs. NCDs are responsible for 59% of the proportional mortality in Bangladesh (% of total deaths in both sexes and all age groups) [8]. Between 1986 and 2006, there has been a nearly nine-fold increase in the proportion of deaths due to NCDs in Matlab, a rural HDSS in Bangladesh [11]. The same HDSS also reported a 3527% increase in mortality due to cardio- and cerebro-vascular diseases during the same period [31]. The death rate from CVDs (cardio vascular diseases) in 2025 is projected to be 21 times the 2003 rate, and Bangladesh is projected to be among the top ten countries in terms of the number of diabetics [11]. The “urban-ness” of diseases such as hypertension, popularly known as “blood pressure”, and diabetes notwithstanding, the prevalence of these diseases is increasing alarmingly among rural communities in Bangladesh. In a cross-sectional study conducted in a Bangladeshi rural community, the prevalence of hypertension among adult males and females was reported to be as high as 31.5 and 29.3%, respectively [32]. In our study, the majority or participants had heard about diabetes and hypertension. Interestingly, the most common sources of information about diabetes and hypertension for the majority of participants were neighbours and not health care providers. Knowledge about the control of diabetes and hypertension was patchy, and respondents reported a variety of control measures. The majority of respondents who reported having diabetes and hypertension were diagnosed through blood/urine tests and blood pressure measurements, respectively. HL is crucial for diseases such as diabetes and hypertension as they require considerable self-care and management, with compliance to treatment being a major issue. Individuals with poorly controlled diabetes and low HL believed that they were optimally controlling their blood glucose, although they did not take measures to improve their glycaemic control [33]. Bangladeshis are among the South Asian populations most likely to have salient NCD behavioural risk factors such as physical inactivity, low intake of fruits and vegetables and tobacco consumption [34]. Many of these risk factors can be modified by adopting a healthy lifestyle. Diabetes and hypertension management include patient involvement in optimizing individual blood glucose and blood pressure levels to prevent complications. It is reported that patients with low HL have difficulty understanding their health care providers’ health communications, are less keen to become involved in medical decision making and are dependent on their family members, friends and health care providers for final decision making [35]. HL is postulated to help diabetics achieve better glycaemic control by influencing their self-efficacy and self-care behaviour [36]. A lack of understanding of the long-term effects of diabetes and hypertension can lead to poor adherence to medication among hypertensive and diabetic patients. In a study conducted in rural China, only 49% of hypertensive respondents knew that they had to take medicine daily. This study also elucidated that most of the rural population received their knowledge about hypertension from village clinics [37]. It is very important point for us to consider these findings from a health systems point of view. Low and middle income countries such as Bangladesh have undergone an epidemiologic transition and face a huge surge in NCDs [7]. The health systems of LMICs (low and middle income countries), which are mostly acute care-oriented, are not prepared to tackle the NCD challenge [7]. A very recent NCD score card ranked Bangladesh’s health system very poorly in terms of NCD preparedness [10]. The Global Strategy for the Prevention and Control of Non Communicable Diseases advocates the combination of primary prevention interventions for whole populations, targeting high-risk individuals and improving access to care [7]. The chronic nature and the expensive, technology-intensive treatment modalities of advanced stage NCDs necessitates focus on primary and secondary prevention efforts [7]. Countries such as Bangladesh will need to come up with a comprehensive health system response to face the enormous challenge posed by NCDs, which will involve finding solutions to many lingering health system shortcomings such as workforce issues, capacity building, referral systems, and patient empowerment, among others [7]. The large number of informal providers practising in rural areas can be explored for this purpose. They can take up health promotion with tailor made messages for the rural population of Bangladesh with low literacy levels. This area requires further in depth exploration in terms of existing disease literacy and the optimum content of messaging for health promotion.

### Limitations

Our study was conducted in a select number of villages in a rural area of Bangladesh and was not based on a nationally representative sample. Thus, the study findings cannot be generalized for the whole country. The association between the dependent and independent variables was assessed through univariate analysis. Thus, the findings do not reflect the nature and magnitude of association between one independent variable and a dependent variable when the effects of other independent variables are controlled for.

## Conclusions

In conclusion, we found that HL is improving in this community with low levels of general literacy. This result was possible because of the large-scale dissemination of health messages through health workers and various media sources promoting science-based health messages, which began a shift from traditional beliefs to modern ones. In Bangladesh, the context-specific promotion of oral rehydration therapy (ORT) for the management of diarrhoea and the aggressive promotion of immunization were of great importance. In Bangladesh, ORT is now used in over 80% [38] of cases of diarrhoea, and immunization coverage by 12 months is now 78% [27], which is a big increase from less than 2% in the mid-eighties [39]. Appropriate HL enhancement programmes need to be undertaken to achieve universal health coverage by tackling the future challenges of hypertension, diabetes and other emerging health threats.

## Abbreviations

BP: Blood pressure
EPI: Expanded Programme on Immunization
HL: Health Literacy
icddr,b: International Centre for Diarrhoeal Disease Research, Bangladesh
LMIC: Low and middle income country
MBBS: Bachelor of Medicine and Bachelor of Surgery
NCD: Non communicable disease
SACMO: Sub assistant community medical officer
